# An Improved Fast Self-Calibration Method for Hybrid Inertial Navigation System under Stationary Condition

**DOI:** 10.3390/s18051303

**Published:** 2018-04-24

**Authors:** Bingqi Liu, Shihui Wei, Guohua Su, Jiping Wang, Jiazhen Lu

**Affiliations:** 1 High-Tech Institute of Xi’an, Xi’an 710025, China; iqgnibuil@126.com; 2 The Science and Technology on Inertial Laboratory, School of Instrumentation Science and Opto-electronics Engineering, Beijing University of Aeronautics and Astronautics, Beijing 100191, China; yunshihui@sina.com (S.W.); suguohua19840522@163.com (G.S.); wjpzj1111@163.com (J.W.)

**Keywords:** hybrid inertial navigation, self-calibration, error models, error parameters

## Abstract

The navigation accuracy of the inertial navigation system (INS) can be greatly improved when the inertial measurement unit (IMU) is effectively calibrated and compensated, such as gyro drifts and accelerometer biases. To reduce the requirement for turntable precision in the classical calibration method, a continuous dynamic self-calibration method based on a three-axis rotating frame for the hybrid inertial navigation system is presented. First, by selecting a suitable IMU frame, the error models of accelerometers and gyros are established. Then, by taking the navigation errors during rolling as the observations, the overall twenty-one error parameters of hybrid inertial navigation system (HINS) are identified based on the calculation of the intermediate parameter. The actual experiment verifies that the method can identify all error parameters of HINS and this method has equivalent accuracy to the classical calibration on a high-precision turntable. In addition, this method is rapid, simple and feasible.

## 1. Introduction

**Figure 1:**
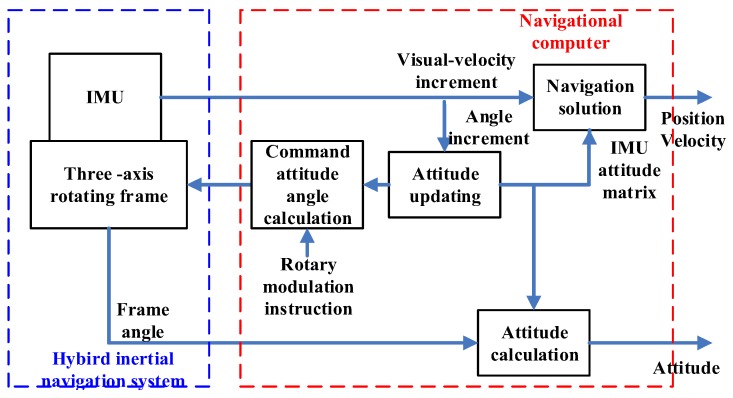
Working principle of the hybrid inertial navigation system.

The inertial navigation system is a high-precision, autonomous navigation system based on gyros and accelerometer measurement. It does not depend on any external system, for example, the platform, station, star, etc. and does not need to send electromagnetic radiation signals to the outside when it is working. It is an indispensable navigation equipment in aerospace, sea and environments with high electro-magnetic interference [1]. According to different construction methods, the INS systems can be divided into platform systems and strapdown systems. The platform-based inertial navigation system, whose inertial devices are installed in a multi-axis stable platform, can achieve high navigation accuracy [2]. However, there are some shortcomings such as complex structure, large volume and weight, poor reliability and high cost. In the strapdown inertial navigation system (SINS), the inertial device and vehicle are connected and there is no longer a physical stable platform. Thus, there are fewer inertial navigation system components and the structure is simpler. This system achieves better volume, weight, cost and reliability while maintaining the system navigation accuracy [3,4] and the preparation time is shortened; however, to further improve the navigation, accuracy is notably difficult [5,6]. In recent years, various research institutions have developed a unique single-axis and dual-axis rotation inertial system, whose accuracy can be improved by approximately one order of magnitude in comparison with the ordinary strapdown inertial navigation system, which indicates that the rotary inertial navigation system has great potential for improving the system accuracy [7,8]. At present, Academic Feng, of Beijing University of Aeronautics and Astronautics, presents a hybrid inertial navigation system (HINS), which combines the features of the existing platform, strapdown and rotary inertial navigation systems. Figure 1 shows the working principle of the hybrid inertial navigation system. In addition to the “self-testing, self-calibration and self-alignment” functions, the hybrid inertial navigation system integrates the physical platform that separates the angular movement of the vehicle, strapdown attitude algorithm and rotation modulation error suppression. In the process, the frame is used to separate the angular movement of the vehicle while performing the rotation modulation to effectively separate and compensate the accelerometer common drift and gyroscopic part error to improve the precision of inertial navigation. It is of great importance to examine a new type of hybrid inertial navigation application [9]. 

Precise calibration of error parameters such as the constant bias, scale factor and misalignment angle of the Fiber Optics Gyros (FOG) and accelerometer is the prerequisite for an inertial navigation system to obtain high-precision navigation performance. The conventional Strap-down Inertial Navigation System (SINS) parameter calibration method can be divided into instrument-level calibration and system-level calibration methods. The instrument-level calibration method includes position and rate calibration, which have the disadvantages of complicated operation, long preparation time and calibration accuracy constrained by turntable accuracy [10,11]. In the system-level calibration, each error term is transformed into a navigation result (position, speed, attitude, etc.) through the navigation solution after the navigation state, which shows the navigation error. The SINS parameters can be estimated if all or part of the navigation error information is obtained. According to different estimation methods, there are the fitting method and the filtering method. It is possible to reduce the dependence of the calibration process on the high-precision turntable [12,13,14,15,16,17,18]. Xiang Gang introduced a once-electrification calibration algorithm for part of the parameters, which can calibrate 18 parameters of the inertial navigation calibration [18]. For the rotary SINS, the system-level calibration method can self-calibrate the system using its own rotation mechanism with the characteristics of rotary inertial navigation. A fast filtering algorithm was proposed by Yuanpei Chen for single-axis rotary inertial navigation, which could calibrate five errors [17]. Zhichao Zheng proposed an eight-position system calibration method to calibrate part of the error of biaxial rotating inertial navigation [16]. Peida Hu proposed a new calibration method for the installation error of the biaxial rotation inertial navigation system, which is based on the attitude error model and attitude difference [19]. The calibration method generally has the problems of high precision for specific parameters, long calibration time for all parameters and relatively low accuracy. With the continuous development of rotating inertial navigation, new calibration methods are constantly introduced and improved. A method to realize the simple, fast and accurate calibration of all parameters of the rotating inertial navigation is the research direction. 

The errors of the three-axis hybrid inertial navigation system can be better isolated through the rotation. The self-calibration of the inertial navigation system can be realized by setting a special rotation strategy and a system-level calibration algorithm [20]. To calibrate the three-axis rotary inertial navigation, many scholars introduced many targeted calibration methods for specific parameters such as the nonlinear error of accelerometer [21], size effect error [22], non-orthogonal angle error of rotating frame [23] and internal lever arm parameters [24]. In the field of full parameter calibration method, Pengyu Gao proposed a least-squares-fitting self-calibration method for three-axis rotary inertial navigation [25]. The calibration of 23 error parameters is completed in three steps, which avoids the cross-coupling effect among different parameters. However, the segmentation calibration results in long calibration time and increases the operation. The method of 19-position calibration proposed by Qingzhong Cai [26] is a more practical high-precision calibration method. The method can calibrate 24 error parameters but the calibration time is slightly long. Weng Hai-na proposed a type of “velocity + position” matching Kalman filtering method [27] based on the discrete calibration for hybrid inertial navigation systems. The method can realize the on-line calibration of inertial navigation system error parameters but the calibration must be based on discrete calibration. In general, there are many good calibration methods for specific parameters to obtain high-precision results. However, for all-parameter high-precision calibration, the long calibration time is a prevalent problem for almost all methods. In this paper, an improved fast-fitting self-calibration method is proposed for hybrid inertial navigation systems. By optimizing the position arrangement, we realize the high precision, quick excitation and identification of the error parameters of the inertial navigation system (INS). Compared with the traditional calibration methods such as the 19-position calibration method, the proposed method has three contributions. First, the calibration time is short: all parameters can be calibrated in 30 min. Second, the calibration process is simple. The high-precision calibration of the inertial navigation parameters can be realized only through the marble plate, which eliminates the dependence on the high-precision turntable. Third, the calibration process is completely automated. The parameter sensitivity of the filtering method is avoided and the self-calibration requirements of the inertial navigation system are satisfied.

This paper is divided into six sections to introduce the improved fast fitting calibration method. The Section 1 is the introduction. The Section 2 introduces the improved hybrid fast calibration fitting method of inertial navigation, calibration method flow, position arrangement, error model, etc. To prove the feasibility of the calibration method, the simulation is performed in the Section 3, which verifies the calibration method. The Section 4 further proves the rapidity and high accuracy of the method by a physical test compared with the traditional 19-position calibration method. The Section 5 summarizes the advantages and features of the improved fast fitting self-calibration method.

## 2. Improved Hybrid Inertial Self-Calibration Method

### 2.1. Calibration Methods and Processes

The calibration method can realize the continuous dynamic self-calibration of full parameters under stationary base conditions. In other words, the gyro and accelerometer data are continuously collected during the rotation of the hybrid inertial axes instead of collecting at only a few fixed positions. Through the system’s position arrangement and intermediate parameter calculation, the least-squares method is used to fit and estimate the error parameter and alignment error of the hybrid inertial navigation. Thus, the calibration of the hybrid inertial navigation can be completed in a single power-on situation. With this method, the hybrid inertial navigation parameters can be quickly self-calibrated with high accuracy and precision, the dependence on the high-precision turntable can be eliminated and the calibration process can be simplified.

For the convenience of narration, first, we introduce several coordinate system definitions:

The Inertial Coordinate Frame ($ox_{i}y_{i}z_{i}$): Its origin is at the center of the earth with *z*-axis parallel to the earth’s rotation direction. The *x*, *y*-axes lie in the earth’s equatorial plane and the *x*-axis points to the First Point of Aries.

The Earth-fixed Coordinate Frame ($ox_{e}y_{e}z_{e}$). Its origin and *z*-axis direction are the same as the inertial frame’s. The *x*, *y*-axes lie in the earth’s equatorial plane with the *x*-axis passing through the main meridian (i.e., longitude = 0).

The Local Geographical Coordinate Frame ($ox_{n}y_{n}z_{n}$): The local level coordinate frame located at the rover is defined as the navigation frame. Its origin at the center of the hybrid inertial navigation system. The *x*-axis is along the local east, the *y*-axis is along local north and the *z*-axis is along the local vertical. 

The IMU Body Coordinate Frame ($ox_{b}y_{b}z_{b}$): Its origin at the center of the hybrid inertial navigation system. The *x*-axis is aligned with the input *x*-axis of the accelerometer. The *y*-axis lies in the plane defined by x and y accelerometer input axis and points to the y accelerometer side, the *z*-axis points upward.

$\theta_{xt}$$\theta_{yt}$$\theta_{zt}$ are the rotation angles, L is the latitude and $\omega_{ie}$ is the autobiographical angular velocity.

The calibration process is as follows:

First, the hybrid inertial navigation system is fixed to the immovable base, so that the outer frame axis Z is located on the horizontal plane (accuracy is +3°) and it points to the south. When the outer frame angle is 0, the middle frame axis is located on a horizontal plane (accuracy is +3°). In other words, in the initial position, the *X* and *Z* axes of the *XYZ* coordinate system are located on the horizontal plane with the precision of +30. At this time, the hybrid inertial rotation angle around the inner ring axis is zero.

Second, the hybrid inertial navigation system rotates into nine positions based on its own three-frame structure. The accuracy of each position relative to the local geo-referenced plane is approximately 3°. During the rotation and each position, the accelerometers and gyro data are continuously collected for approximately 3 min. The collected data include the output of the table and gyro.

Third, non-real-time calculations of hybrid inertial navigation system parameters are conducted by processing the collected data.

**Figure 2:**
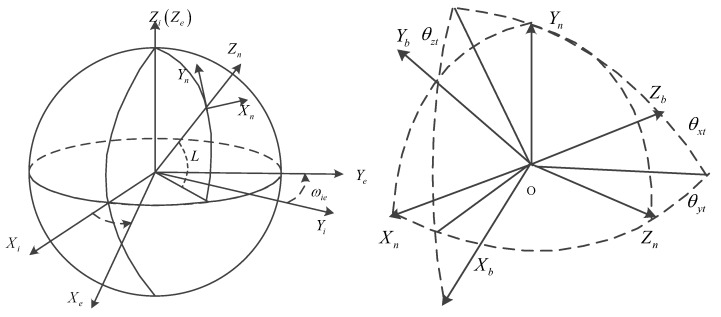
The coordinate frames used in this paper.

**Figure 3:**
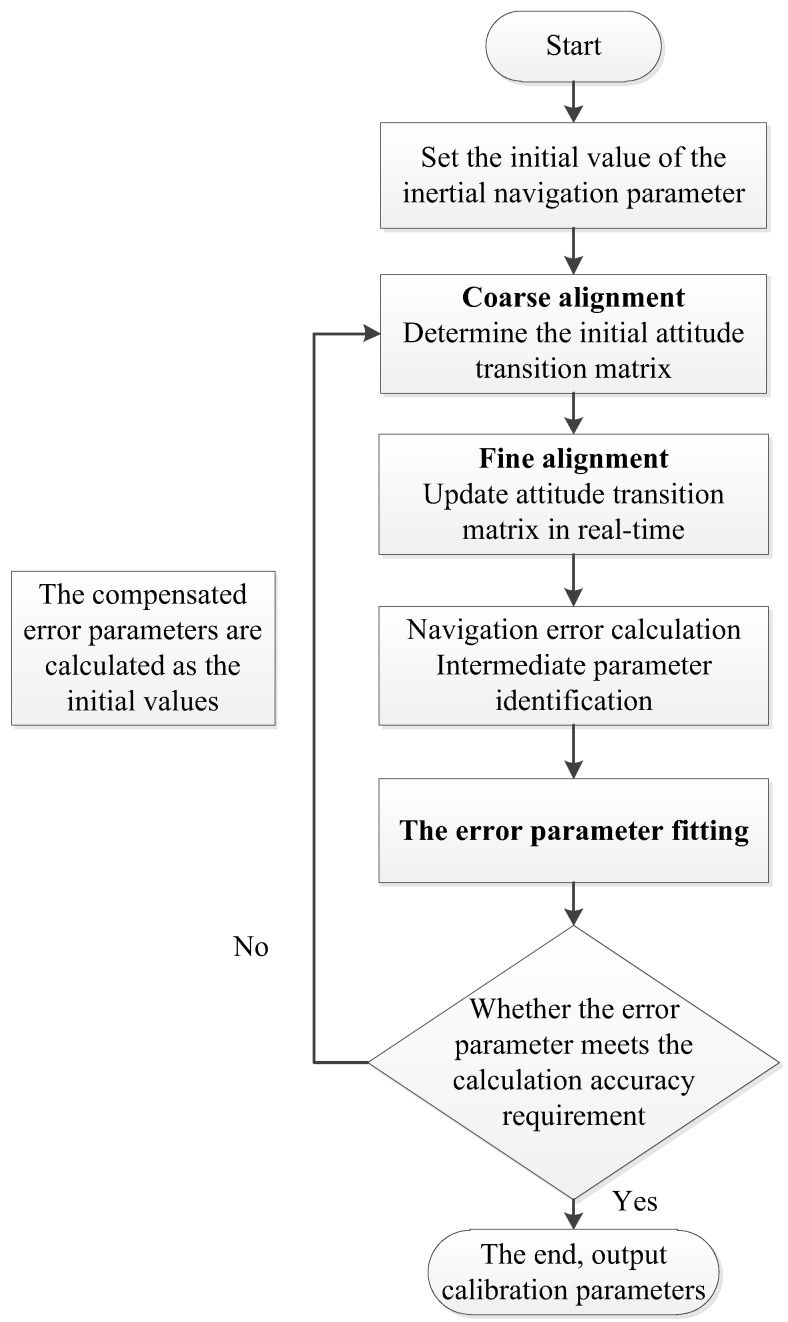
Self-calibration method process.

The specific implementation process is shown in Figure 2 and the parameter calibration accuracy can be further improved through a step-by-step iterative approach. The Self-calibration method process is shown in Figure 3.

### 2.2. The Calibration Rotation Sequence

The inertial analysis and alignment algorithm is used to obtain the initial position of the self-alignment. Then, according to the pre-designed multi-position flip order of rotation, the inertial instrumentation error parameters can be excited in the entire process of rotation and rest. The navigation output speed and course information are measured and stored as the navigation error observation. Finally, the least-squares method is used to separate the error parameters.

**Figure 4:**
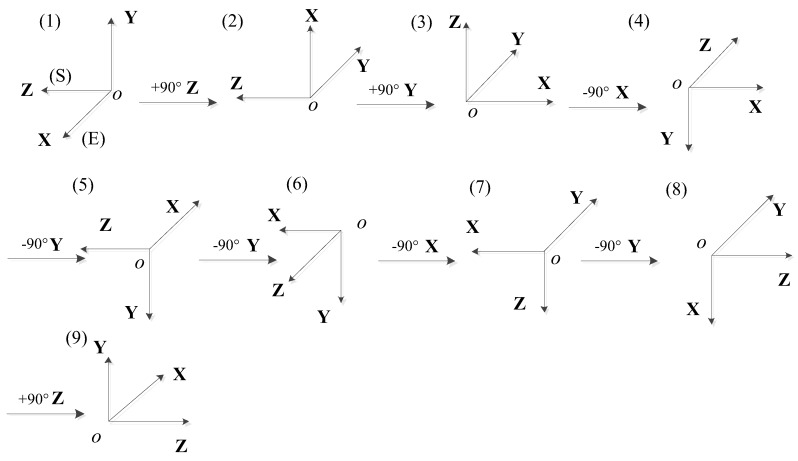
Rotation sequence.

During calibration, the hybrid inertial navigation system rotates nine positions based on its own three-frame structure. The starting position *X* and *Z* axes are in the horizontal plane and the *Y* axis points upward. The principle and layout of the calibration are that each parameter can be fully excited and each error parameter can be separated while minimizing the cross-coupling effects among the error terms. The specific rotation order is shown in Figure 4 and Table 1.

### 2.3. Fitting Method Research

#### 2.3.1. Inertial Navigation System Error Model

Using the propagation characteristics of the inertial navigation system’s error parameters during navigation, the inertial measurement unit (IMU) is flipped and stopped by rotating or stationary frames to excite different parameters. When the acquired data enable the parameters of interest to be observed, the parameter error of the inertial navigation system can be estimated. The high-precision inertial navigation system parameters can be obtained using iterative calculation to make the error of the inertial navigation system converge to nearly zero.

The error mathematical model of the acceleration measurement channel is:

(1) $$\delta f^{b}=\left[\begin{array}{c} \delta f_{x}^{b}\\ \delta f_{y}^{b}\\ \delta f_{z}^{b} \end{array}\right]=\left[\begin{array}{ccc} K_{axx} & 0 & 0\\ K_{ayx} & K_{ayy} & 0\\ K_{azx} & K_{azy} & K_{azz} \end{array}\right]\left[\begin{array}{c} f_{x}^{b}\\ f_{y}^{b}\\ f_{z}^{b} \end{array}\right]+\left[\begin{array}{c} \eta_{0x}\\ \eta_{0y}\\ \eta_{0z} \end{array}\right]$$

In the equation, $f_{}^{b}$ is the ideal value, $\delta f^{b}$ is the accelerometer measurement error, $\delta f_{I}^{b}(I=x,y,z)$ is the acceleration measurement channel error, $K_{aII}$ (I=x,y,z) is the accelerometer scale factor error, $\eta_{I}$ (I=x,y,z) is the acceleration measurement channel bias and $K_{aIJ}$ (I=x,y,z; J=x,y,z; I≠J) is the acceleration measurement channel installation error parameter.

The error mathematical model of the angular velocity measurement channel is:

(2) $$\delta \omega_{ib}^{b}=\left[\begin{array}{c} \delta \omega_{ibx}^{b}\\ \delta \omega_{iby}^{b}\\ \delta \omega_{ibz}^{b} \end{array}\right]=\left[\begin{array}{ccc} W_{gxx} & W_{gxy} & W_{gxz}\\ W_{gyx} & W_{gyy} & W_{gyz}\\ W_{gzx} & W_{gzy} & W_{gzz} \end{array}\right]\left[\begin{array}{c} \omega_{ibx}^{b}\\ \omega_{iby}^{b}\\ \omega_{ibz}^{b} \end{array}\right]+\left[\begin{array}{c} \omega_{0x}\\ \omega_{0y}\\ \omega_{0z} \end{array}\right]$$

In the equation, $\omega_{ib}^{b}$ is the ideal value, $\delta \omega_{I}^{b}$ is the angular velocity measurement channel error, $W_{gII}$ (I=x,y,z) is the angular velocity measurement channel scaling factor, $\omega_{0I}$ (I=x,y,z) is the angular velocity measurement channel constant drift and $W_{gIJ}$ (I=x,y,z;J=x,y,z;I≠J) is the angular velocity measurement channel installation error parameter.

#### 2.3.2. Systematic Error Model

First, the accelerometer and gyros data are used to align the inertial navigation system. After the alignment is completed, navigation begins from the first position; the navigation speed error $\delta {\dot{v}}^{n}$ and heading error $\dot{\varphi }$ are measured and recorded as the navigation error observation.

The speed error model is:

(3) $$\begin{array}{l} \delta {\dot{v}}^{n}=f^{n}\times \dot{\varphi }+C_{b}^{n}\delta f^{b}-(2\omega_{ie}^{n}+\omega_{en}^{n})\times \delta v^{n}\\ -(2\delta \omega_{ie}^{n}+\delta \omega_{en}^{n})\times v^{n} \end{array}$$

The attitude error model is:

(4) $${\dot{\varphi }}^{n}=-\omega_{in}^{n}\times \varphi^{n}+\delta \omega_{in}^{n}-C_{b}^{n}\delta \omega_{ib}^{b}$$

In the equation:

i is the inertial coordinate system; e is the earth coordinate system; n is the navigation coordinate system; b is the strapdown inertial combined coordinate system; and $\varphi^{n}$ is the misalignment angle between the calculated coordinate system and the ideal navigation coordinate system, that is, the inertial navigation attitude error angle. ω and δω are the angular velocity and angular velocity error; the superscript indicates the component values in the corresponding coordinate system and the subscripts represent the relative motion of the coordinate system. v and δv are the speed and velocity error; f and δf are the specific force and force error; and $C_{b}^{n}$ is the attitude matrix.

According to the pre-arranged rotation sequence, each position of the stationary and dynamic flip data can be continuously sampled. The error parameters can be iteratively calculated using the local gravity and latitude information with the origin of navigation, speed error and attitude error formula.

In the calibration process, regardless of the random error of the inertial instrument, when the system is rotated from one position to another, that is, when the system is in the stationary-roll-stationary motion, the error of the stationary navigation speed $\delta v^{n}$ in a short time after the turning position is mainly caused by the inertia instrument errors $\delta f^{b}$ and $\delta \omega_{ib}^{b}$. Considering the error model of the acceleration measurement channel and angular velocity measurement channel, we can assume that the system stationary-roll-stationary motion excites part of the inertial instrument error parameters. If the direction and angle of rotation are different, the error parameters of the inertial meter are different. All error parameters of the inertial instrument can be redundantly excited by turning the position.

The velocity error formula in the navigation coordinate system (n) is shown in Equation (3); the analytical expressions of $\omega_{ie}^{n}$ and $\omega_{en}^{n}$ are as follows:

(5) $$\left\{ \begin{array}{l} \omega_{ie}^{n}={\left[\begin{array}{ccc} 0 & \omega_{ie}^{}\cos L & \omega_{ie}^{}\sin L \end{array}\right]}^{T}\\ \omega_{en}^{n}={\left[\begin{array}{ccc} -\frac{V_{N}}{R_{M}+h} & \frac{V_{E}}{R_{N}+h} & \frac{V_{E}\tan L}{R_{N}+h} \end{array}\right]}^{T} \end{array}\right.$$

The calculation error of Equation (5) is as follows:

(6) $$\left\{ \begin{array}{l} \delta \omega_{ie}^{n}={\left[\begin{array}{ccc} 0 & -\delta L\omega_{ie}^{}\sin L & \delta L\omega_{ie}^{}\cos L \end{array}\right]}^{T}\\ \delta \omega_{en}^{n}=\left[\begin{array}{c} -\frac{\delta V_{N}}{R_{M}+h}+\frac{\delta h\cdot V_{N}}{{\left(R_{M}+h\right)}^{2}}\\ \frac{\delta V_{E}}{R_{N}+h}-\frac{\delta h\cdot V_{E}}{{\left(R_{N}+h\right)}^{2}}\\ \frac{\tan L\cdot \delta V_{E}}{R_{N}+h}+\frac{\delta L\cdot V_{E}\cdot \sec^{2}L}{R_{N}+h}-\frac{\delta h\cdot V_{E}\cdot \sec L}{{\left(R_{N}+h\right)}^{2}} \end{array}\right] \end{array}\right.$$

In the equation, $R_{M}$ and $R_{N}$ are the radius of curvature in meridian and radius of curvature in unitary, respectively; $\delta V_{N}$ and $\delta V_{E}$ are the navigation speed errors, *L* is the geodetic latitude, *h* is the altitude.

In each calibration position, the inertia navigation system is quasi-stationary; $V_{N}$ and $V_{E}$ do not change and can be approximated as 0. Simultaneously, $\delta v^{n}$, $\delta \omega_{ie}^{n}$ and $\delta \omega_{en}^{n}$ are also 0.

We substitute Equations (5) and (6) into Equation (3) and rewrite (3) as:

(7) $$\delta {\dot{v}}^{n}=f^{n}\times \varphi^{n}+C_{b}^{n}\delta f^{b}-(2\omega_{ie}^{n})\times \delta v^{n}+\nabla^{n}$$

The speed increment $V_{igk}{}^{\left(j\right)}$ and rotation angle increment $\theta_{k}{}^{\left(j\right)}$ of each calibration position can be calculated according to the accelerometer and gyro output in the calibration process (j is the calibration position and k is the sampling time). Based on the real-time update calculation of the attitude transition matrix, the speed error is calculated according to the output information of the calibration and Equation (7) is rewritten as:

(8) $$\begin{array}{cc} {V_{nk}}^{\left(j\right)} & =V_{nk-1}{}^{\left(j\right)}+\frac{1}{2}(C_{nbk}+C_{nbk-1})f_{k}\\ & -\left(\begin{array}{c} 2\omega_{ie}^{}\sin LV_{znk-1}{}^{\left(j\right)}\\ g-2\omega_{ie}^{}\cos LV_{znk-1}{}^{\left(j\right)}\\ 2\omega_{ie}^{}\cos LV_{ynk-1}{}^{\left(j\right)}-2\omega_{ie}^{}\sin LV_{xnk-1}{}^{\left(j\right)} \end{array}\right)\Delta t \end{array}$$

Δt is the time interval of the inertial navigation system, $C_{nbk}$ is attitude transformation matrix between coordinate frame *n* and another frame *b*. Among them:

(9) $$f_{k}=\left(\begin{array}{c} f_{xk}\\ f_{yk}\\ f_{zk} \end{array}\right)=\left(\begin{array}{c} b_{xk}K_{axx}-\eta_{0x}\Delta t\\ b_{yk}K_{ayy}-b_{xk}K_{axx}k_{ayx}-\eta_{0y}\Delta t\\ b_{zk}K_{azz}-b_{xk}K_{axx}K_{azx}-b_{yk}K_{ayy}K_{azy}-\eta_{0z}\Delta t \end{array}\right)$$

$b_{xk},b_{yk},b_{zk}$ are the accelerometer read data of step kth.

#### 2.3.3. Attitude Updated Equation

The attitude transformation matrix model is as follows:

(10) $$C_{nbk}=C_{ni}C_{nbk-1}C_{ib}(k=1,\ldots ,N);C_{nb0}=C_{nb}(0)$$

The initial position of the inertial transformation matrix is $C_{nb0}{}^{\left(0\right)}$:

(11) $$C_{nik}=\left(\begin{array}{ccc} \cos^{2}L+\sin^{2}L\cos(\omega_{ie}\Delta tk^{\prime }) & \sin L\cos L[1-\cos(\omega_{ie}\Delta tk^{\prime })] & -\sin L\sin(\omega_{ie}\Delta tk^{\prime })\\ \sin L\cos L[1-\cos(\omega_{ie}\Delta tk^{\prime })] & \sin^{2}L+\cos^{2}L\cos(\omega_{ie}\Delta tk^{\prime }) & \cos L\sin(\omega_{ie}\Delta tk^{\prime })\\ \sin L\sin(\omega_{ie}\Delta tk^{\prime }) & -\cos L\sin(\omega_{ie}\Delta tk^{\prime }) & \cos(\omega_{ie}\Delta tk^{\prime }) \end{array}\right)$$

(12) $$\left\{ \begin{array}{l} C_{ib}=\left(\begin{array}{ccc} 1-k_{2}(p_{y}{}^{2}+p_{z}{}^{2}) & -k_{1}p_{z}+k_{2}p_{x}p_{y} & k_{1}p_{y}+k_{2}p_{x}p_{z}\\ k_{1}p_{z}+k_{2}p_{x}p_{y} & 1-k_{2}(p_{x}{}^{2}+p_{z}{}^{2}) & -k_{1}p_{x}+k_{2}p_{z}p_{y}\\ -k_{1}p_{y}+k_{2}p_{x}p_{z} & k_{1}p_{x}+k_{2}p_{z}p_{y} & 1-k_{2}(p_{y}{}^{2}+p_{x}{}^{2}) \end{array}\right)\\ k_{1}=1-\left(p_{x}{}^{2}+p_{y}{}^{2}+p_{z}{}^{2}\right)/6\\ k_{2}=0.5[1+\left(p_{x}{}^{2}+p_{y}{}^{2}+p_{z}{}^{2}\right)/12] \end{array}\right.$$

A single-sample iterative coning compensation algorithm is used to iteratively calculate the rotation vector as:

(13) $$\left\{ \begin{array}{l} \delta p_{i(k)}=\left(\Delta \theta_{i(k-1)}\times \Delta \theta_{i(k)}\right)/12\\ p_{i(k)}=\theta_{ik}+\delta p_{i(k)} \end{array}\right.$$

$\theta_{ik}$ is the increment of the k step attitude angle of the gyro and can be calculated according to the gyro pulse output of step k. To improve the accuracy and convergence speed of the attitude transition matrix, the angular increment is calculated from the pulse output information in two steps. First, the effect of zero bias is deducted. Then, we compensate the installation error. The calculation formula is as follows:

(14) $$\left\{ \begin{array}{l} o_{xd}=o_{xk}W_{gxx}-D_{0x}\Delta t\\ o_{yd}=o_{yk}W_{gyy}-D_{0}{}_{y}\Delta t\\ o_{zd}=o_{zk}W_{gzz}-D_{0}{}_{z}\Delta t \end{array}\right.$$

(15) $$\left\{ \begin{array}{l} \theta_{xk}=o_{xd}-(K_{gxy}o_{yd}+K_{gxz}o_{zd})\\ \theta_{yk}=o_{yd}-(K_{gyx}o_{xd}+K_{gyz}o_{zd})\\ \theta_{zk}=o_{zd}-(K_{gzx}o_{xd}+K_{gzy}o_{yd}) \end{array}\right.$$

$o_{xk},o_{yk},o_{zk}$ are the gyro collection data of step k.

#### 2.3.4. Intermediate Parameter Identification

The uncompensated accelerometer error $\Delta_{xn}{}^{\left(j\right)},\Delta_{yn}{}^{\left(j\right)},\Delta_{zn}{}^{\left(j\right)}$ (j=1,…,9) and gyro drift $\omega_{xn}{}^{\left(j\right)},\omega_{yn}{}^{\left(j\right)},\omega_{zn}{}^{\left(j\right)}$ of each calibration position in the local geographic coordinate system are calculated as follows:

(16) $$\left\{ \begin{array}{cc} {\Delta_{xn}}^{\left(j\right)} & =q_{21}\sum_{m=m_{b}^{\left(j\right)}+1}^{m_{e}^{\left(j\right)}}\left({V_{nxm}}^{\left(j\right)}-{V_{bx}}^{\left(j\right)}\right)+q_{22}g\Delta t\sum_{m=m_{b}^{\left(j\right)}+1}^{m_{e}^{\left(j\right)}}\left({V_{nxm}}^{\left(j\right)}-{V_{bx}}^{\left(j\right)})(m-m_{b}^{\left(j\right)}\right)\\ & +q_{23}\frac{g\Delta t^{2}}{2}\sum_{m=m_{b}^{\left(j\right)}+1}^{m_{e}^{\left(j\right)}}({V_{nxm}}^{\left(j\right)}-{V_{bx}}^{\left(j\right)}){\left(m-m_{b}^{\left(j\right)}\right)}^{2}\\ {\Delta_{yn}}^{\left(j\right)} & =(-\sum_{m=m_{b}^{\left(j\right)}+1}^{m_{e}^{\left(j\right)}}\left({V_{nym}}^{\left(j\right)}-{V_{by}}^{\left(j\right)}\right)+2\frac{\sum_{m=m_{b}^{\left(j\right)}+1}^{m_{e}^{\left(j\right)}}\left({V_{nym}}^{\left(j\right)}-{V_{by}}^{\left(j\right)})(m-m_{b}^{\left(j\right)}\right)}{N^{\left(j\right)}+1})/(N^{\left(j\right)}(N^{\left(j\right)}-1)g\Delta t)\\ {\Delta_{zn}}^{\left(j\right)} & =q_{21}\sum_{m=m_{b}^{\left(j\right)}+1}^{m_{e}^{\left(j\right)}}\left({V_{nzm}}^{\left(j\right)}-{V_{bz}}^{\left(j\right)}\right)+q_{22}g\Delta t\sum_{m=m_{b}^{\left(j\right)}+1}^{m_{e}^{\left(j\right)}}\left({V_{nzm}}^{\left(j\right)}-{V_{bz}}^{\left(j\right)})(m-m_{b}^{\left(j\right)}\right)\\ & +q_{23}\frac{g\Delta t^{2}}{2}\sum_{m=m_{b}^{\left(j\right)}+1}^{m_{e}^{\left(j\right)}}({V_{nzm}}^{\left(j\right)}-{V_{bz}}^{\left(j\right)}){\left(m-m_{b}^{\left(j\right)}\right)}^{2} \end{array}\right.$$

(17) $$\left\{ \begin{array}{cc} {\omega_{xn}}^{\left(j\right)}= & q_{31}\sum_{m=m_{b}^{\left(j\right)}+1}^{m_{e}^{\left(j\right)}}\left(V_{nxm}{}^{\left(j\right)}-V_{bx}{}^{\left(j\right)}\right)+q_{32}g\Delta t\sum_{m=m_{b}^{\left(j\right)}+1}^{m_{e}^{\left(j\right)}}\left(V_{nxm}{}^{\left(j\right)}-V_{bx}{}^{\left(j\right)})(m-m_{b}^{\left(j\right)}\right)\\ & +q_{33}\frac{g\Delta t^{2}}{2}\sum_{m=m_{b}^{\left(j\right)}+1}^{m_{e}^{\left(j\right)}}(V_{nxm}{}^{\left(j\right)}-V_{bx}{}^{\left(j\right)}){\left(m-m_{b}^{\left(j\right)}\right)}^{2}\\ {\omega_{yn}}^{\left(j\right)}= & \left(-\sum_{m=m_{b}^{\left(j\right)}+1}^{m_{e}^{\left(j\right)}}\theta_{m}{}^{\left(j\right)}-2\frac{\sum_{m=m_{b}^{\left(j\right)}+1}^{m_{e}^{\left(j\right)}}(m-m_{b}^{\left(j\right)})\theta_{m}{}^{\left(j\right)}}{N^{\left(j\right)}+1})/(N^{\left(j\right)}(N^{\left(j\right)}-1)\Delta t\right)\\ {\omega_{zn}}^{\left(j\right)}= & q_{31}\sum_{m=m_{b}^{\left(j\right)}+1}^{m_{e}^{\left(j\right)}}\left(V_{nzm}{}^{\left(j\right)}-V_{bz}{}^{\left(j\right)}\right)+q_{32}g\Delta t\sum_{m=m_{b}^{\left(j\right)}+1}^{m_{e}^{\left(j\right)}}\left(V_{nzm}{}^{\left(j\right)}-V_{bz}{}^{\left(j\right)})(m-m_{b}^{\left(j\right)}\right)\\ & +q_{33}\frac{g\Delta t^{2}}{2}\sum_{m=m_{b}^{\left(j\right)}+1}^{m_{e}^{\left(j\right)}}(V_{nzm}{}^{\left(j\right)}-V_{bz}{}^{\left(j\right)}){\left(m-m_{b}^{\left(j\right)}\right)}^{2} \end{array}\right.$$

In the equation, when $m=m_{b}{}^{\left(j\right)}$ and $N^{\left(j\right)}=m_{e}{}^{\left(j\right)}-m_{b}{}^{\left(j\right)}$, $V_{bx}{}^{\left(j\right)},V_{by}{}^{\left(j\right)},V_{bz}{}^{\left(j\right)}$ align with $V_{gxm}{}^{\left(j\right)},V_{gym}{}^{\left(j\right)},V_{gzm}{}^{\left(j\right)}$. $q_{21},q_{22},q_{23},q_{31},q_{32},q_{33}$ are the elements of matrix $Q=A^{-1}$.

(18) $$A=\left(\begin{array}{ccc} N^{\left(j\right)} & g\Delta tS_{1} & \frac{g\Delta t^{2}}{2}S_{2}\\ g\Delta tS_{1} & g^{2}\Delta t^{2}S_{2} & \frac{g^{2}\Delta t^{3}}{2}S_{3}\\ \frac{g\Delta t^{2}}{2}S_{2} & \frac{g^{2}\Delta t^{3}}{2}S_{3} & \frac{g^{2}\Delta t^{4}}{4}S_{4} \end{array}\right)$$

(19) $$\begin{array}{l} S_{1}=N^{\left(j\right)}(N^{\left(j\right)}+1)/2\\ S_{2}=N^{\left(j\right)}(N^{\left(j\right)}+1)(2N^{\left(j\right)}+1)/6\\ S_{3}=N^{(j)2}{\left(N^{\left(j\right)}+1\right)}^{2}/4\\ S_{4}=N^{\left(j\right)}(N^{\left(j\right)}+1)(2N^{\left(j\right)}+1)(3N^{(j)2}+3N^{\left(j\right)}-1)/30 \end{array}$$

#### 2.3.5. Systematic Error Parameters Determination and Compensation

Based on the identification of the intermediate parameters, 21 systematic error parameters to be calibrated are identified through algebraic relationships with the systematic error parameters. The 21 systematic error parameters are scaled as follows:

Accelerometer bias:

(20) $$\begin{array}{l} {\hat{\eta }}_{0x}=\left(\begin{array}{l} 8\Delta_{xn}{}^{\left(1\right)}+\Delta_{xn}{}^{\left(4\right)}-4\Delta_{xn}{}^{\left(5\right)}+\Delta_{xn}{}^{\left(6\right)}-6\Delta_{xn}{}^{\left(9\right)}\\ -6\Delta_{zn}{}^{\left(3\right)}-5\Delta_{zn}{}^{\left(4\right)}+5\Delta_{zn}{}^{\left(6\right)}+6\Delta_{zn}{}^{\left(7\right)} \end{array}\right)g/40\\ {\hat{\eta }}_{0y}=\left(\begin{array}{l} 16\Delta_{xn}{}^{\left(1\right)}-20\Delta_{xn}{}^{\left(2\right)}-20\Delta_{xn}{}^{\left(3\right)}+17\Delta_{xn}{}^{\left(4\right)}+12\Delta_{xn}{}^{\left(5\right)}+17\Delta_{xn}{}^{\left(6\right)}-20\Delta_{xn}{}^{\left(7\right)}\\ -20\Delta_{xn}{}^{\left(8\right)}+18\Delta_{xn}{}^{\left(9\right)}-2\Delta_{zn}{}^{\left(3\right)}-5\Delta_{zn}{}^{\left(4\right)}+5\Delta_{zn}{}^{\left(6\right)}+2\Delta_{zn}{}^{\left(7\right)} \end{array}\right)g/80\\ {\hat{\eta }}_{0z}=\left(\begin{array}{l} -6\Delta_{xn}{}^{\left(4\right)}+6\Delta_{xn}{}^{\left(6\right)}+8\Delta_{zn}{}^{\left(1\right)}+7\Delta_{zn}{}^{\left(2\right)}-2\Delta_{zn}{}^{\left(4\right)}\\ +4\Delta_{zn}{}^{\left(5\right)}-2\Delta_{zn}{}^{\left(6\right)}-7\Delta_{zn}{}^{\left(8\right)}-8\Delta_{zn}{}^{\left(9\right)} \end{array}\right)g/46 \end{array}$$

Accelerometer scale factor:

(21) $$\begin{array}{l} {\hat{K}}_{axx}=\left(\Delta_{yn}{}^{\left(2\right)}+\Delta_{yn}{}^{\left(8\right)}\right)/2\\ {\hat{K}}_{ayy}=\left(3\Delta_{yn}{}^{\left(1\right)}+2\Delta_{yn}{}^{\left(4\right)}+2\Delta_{yn}{}^{\left(5\right)}+2\Delta_{yn}{}^{\left(6\right)}+3\Delta_{yn}{}^{\left(9\right)}\right)/12\\ {\hat{K}}_{azz}=\left(\Delta_{yn}{}^{\left(3\right)}+\Delta_{yn}{}^{\left(7\right)}\right)/2 \end{array}$$

Installation error of the accelerometer:

(22) $$\begin{array}{l} {\hat{K}}_{ayx}=\left(\begin{array}{l} 16\Delta_{xn}{}^{\left(1\right)}-40\Delta_{xn}{}^{\left(2\right)}-3\Delta_{xn}{}^{\left(4\right)}+12\Delta_{xn}{}^{\left(5\right)}-3\Delta_{xn}{}^{\left(6\right)}+40\Delta_{xn}{}^{\left(8\right)}\\ -22\Delta_{xn}{}^{\left(9\right)}-4\Delta_{zn}{}^{\left(3\right)}+15\Delta_{zn}{}^{\left(4\right)}-15\Delta_{zn}{}^{\left(6\right)}+4\Delta_{zn}{}^{\left(7\right)} \end{array}\right)/80\\ {\hat{K}}_{azx}=\left(\Delta_{zn}{}^{\left(2\right)}-\Delta_{zn}{}^{\left(3\right)}-\Delta_{zn}{}^{\left(7\right)}+\Delta_{zn}{}^{\left(8\right)}\right)/2\\ {\hat{K}}_{azy}=\left(\begin{array}{l} -23\Delta_{xn}{}^{\left(3\right)}+9\Delta_{xn}{}^{\left(4\right)}-9\Delta_{xn}{}^{\left(6\right)}+23\Delta_{xn}{}^{\left(7\right)}+11\Delta_{zn}{}^{\left(1\right)}+\Delta_{zn}{}^{\left(2\right)}\\ +3\Delta_{zn}{}^{\left(4\right)}-6\Delta_{zn}{}^{\left(5\right)}+3\Delta_{zn}{}^{\left(6\right)}-\Delta_{zn}{}^{\left(8\right)}-11\Delta_{zn}{}^{\left(9\right)} \end{array}\right)/46 \end{array}$$

Gyros zero-drift:

(23) $$\begin{array}{l} {\hat{\omega }}_{0x}=\left(\begin{array}{l} 62\omega_{xn}{}^{\left(1\right)}+3\omega_{xn}{}^{\left(2\right)}+3\omega_{xn}{}^{\left(3\right)}+5.5\omega_{xn}{}^{\left(4\right)}-34\omega_{xn}{}^{\left(5\right)}+5.5\omega_{xn}{}^{\left(6\right)}+3\omega_{xn}{}^{\left(7\right)}\\ +3\omega_{xn}{}^{\left(8\right)}-51\omega_{xn}{}^{\left(9\right)}+3\omega_{yn}{}^{\left(1\right)}+48\omega_{yn}{}^{\left(2\right)}-2\omega_{yn}{}^{\left(4\right)}-2\omega_{yn}{}^{\left(5\right)}-2\omega_{yn}{}^{\left(6\right)}\\ -48\omega_{yn}{}^{\left(8\right)}+3\omega_{yn}{}^{\left(9\right)}-48\omega_{zn}{}^{\left(3\right)}-39.5\omega_{zn}{}^{\left(4\right)}+39.5\omega_{zn}{}^{\left(6\right)}+48\omega_{zn}{}^{\left(7\right)} \end{array}\right)/418\\ {\hat{\omega }}_{0y}=\left(\begin{array}{l} 25\omega_{xn}{}^{\left(1\right)}-32.5\omega_{xn}{}^{\left(2\right)}-32.5\omega_{xn}{}^{\left(3\right)}+27.5\omega_{xn}{}^{\left(4\right)}+20\omega_{xn}{}^{\left(5\right)}+27.5\omega_{xn}{}^{\left(6\right)}-32.5\omega_{xn}{}^{\left(7\right)}\\ -32.5\omega_{xn}{}^{\left(8\right)}+30\omega_{xn}{}^{\left(9\right)}+72\omega_{yn}{}^{\left(1\right)}+2.5\omega_{yn}{}^{\left(2\right)}-48\omega_{yn}{}^{\left(4\right)}-48\omega_{yn}{}^{\left(5\right)}-48\omega_{yn}{}^{\left(6\right)}\\ -2.5\omega_{yn}{}^{\left(8\right)}+72\omega_{yn}{}^{\left(9\right)}-2.5\omega_{zn}{}^{\left(3\right)}-7.5\omega_{zn}{}^{\left(4\right)}+7.5\omega_{zn}{}^{\left(6\right)}+2.5\omega_{zn}{}^{\left(7\right)} \end{array}\right)/418\\ {\hat{\omega }}_{0z}=\left(-4\omega_{xn}{}^{\left(4\right)}+4\omega_{xn}{}^{\left(6\right)}+5\omega_{yn}{}^{\left(3\right)}-5\omega_{yn}{}^{\left(7\right)}+6\omega_{zn}{}^{\left(1\right)}+5\omega_{zn}{}^{\left(2\right)}+4\omega_{zn}{}^{\left(5\right)}-5\omega_{zn}{}^{\left(8\right)}-6\omega_{zn}{}^{\left(9\right)}\right)/44 \end{array}$$

Gyros scale factor:

(24) $$\begin{array}{l} {\hat{W}}_{gxx}=(\Delta_{zn}{}^{\left(4\right)}-\Delta_{zn}{}^{\left(7\right)})/\pi \\ {\hat{W}}_{gyy}=(\Delta_{xn}{}^{\left(8\right)}-\Delta_{xn}{}^{\left(3\right)})/\pi \\ {\hat{W}}_{gzz}=(\Delta_{zn}{}^{\left(2\right)}-\Delta_{zn}{}^{\left(9\right)})/\pi \end{array}$$

Installation error of the accelerometer:

(25) $$\begin{array}{l} {\hat{W}}_{gxz}=(\Delta_{xn}{}^{\left(2\right)}-\Delta_{xn}{}^{\left(9\right)})/2;\\ {\hat{W}}_{gyz}=-(\Delta_{xn}{}^{\left(2\right)}+\Delta_{xn}{}^{\left(9\right)})/2;\\ {\hat{W}}_{gzx}=(\Delta_{xn}{}^{\left(4\right)}-\Delta_{xn}{}^{\left(7\right)})/2;\\ {\hat{W}}_{gyx}=(\Delta_{xn}{}^{\left(4\right)}+\Delta_{xn}{}^{\left(7\right)})/2;\\ {\hat{W}}_{gxy}=(s+d)/2;\\ {\hat{W}}_{gzy}=(s-d)/2;\\ (s=(\Delta_{xn}{}^{\left(5\right)}-\Delta_{zn}{}^{\left(6\right)})/2,\\ d=(\Delta_{xn}{}^{\left(6\right)}-\Delta_{zn}{}^{\left(3\right)}+\Delta_{zn}{}^{\left(5\right)}-\Delta_{zn}{}^{\left(8\right)})/4 \end{array}$$

The original 21 parameters are compensated after calculating the uncompensated error parameters. This process is repeated until the desired precision is attained.

## 3. Mathematical Simulation

### 3.1. Simulation Method and Process

**Figure 5:**
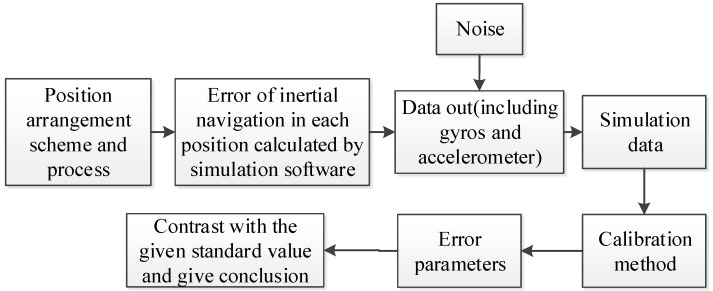
Mathematical simulation sketch map.

To verify the effectiveness of the proposed method, a simulation environment is built under all-digital conditions and the method is simulated. First, the simulation calculation software is input according to the proposed position arrangement scheme. The software simulates the state of inertial navigation system at each position. The accelerometer and gyros pulse data of the hybrid inertial navigation system in self-calibration are mathematically simulated. The fast-fitting calibration algorithm is used to process the data. Finally, various error parameters of each inertial device can be calibrated. The sketch map of mathematical simulation is shown in Figure 5.

### 3.2. Simulation Results and Conclusions

The hybrid inertial navigation system fast self-calibration method is used to comprehensively and accurately calibrate the systematic error parameters. The data results are shown in Table 2.

First, a standard value is provided to 21 error parameters such as the gyro zero drift, accelerometer zero bias, scale factor and installation error. Then, the outputs of the gyros and accelerometers are generated by mathematical simulation. The simulation is performed according to the position arrangement and random noise was added to the data results. The calibration algorithm was used to fit the data. Finally, the simulation results of 21 error parameters were obtained. The results of the simulation are compared with the given standard values to obtain the calibration accuracy. The accuracy requirements of three directions of the gyro zero-drift errors are less than 3.0 × 10^−^^2^, the scale factor error is less than 1 ppm and the installation error is ≤15 arcsec. The accelerometer zero-bias errors in three directions are less than 5.0 × 10^−^^5^. The error of the scale factor is less than 1 ppm and the installation error is less than 10″. The calibration accuracy obtained by the simulation is shown in Table 2. The maximum gyro zero drift error is 0.00406°/h, the maximum error of gyro scale factor is 0.93 ppm and the maximum error of gyro installation error is 7.3″. The maximum accelerometer zero-bias error is 7.03 × 10^−6^ g, the maximum error of the accelerometer scale factor is 0.75 ppm and the maximum error of accelerometer installation is 5.2″. The calibration precision of all parameters satisfies the accuracy requirements. In addition, the entire calibration process is completed within 30 min and the speed is fast. The simulation data show that the fast fitting calibration method can finish the calibration of all 21 error parameters with the error within the requirements and with high precision.

## 4. Experimental Verification

To verify the feasibility and accuracy of the hybrid inertial navigation system calibration method, the hybrid inertial navigation system was calibrated in a physical calibration experiment and compared with the 19-position calibration method on high precision turntable [26].

### 4.1. Experimental Scheme

**Figure 6:**
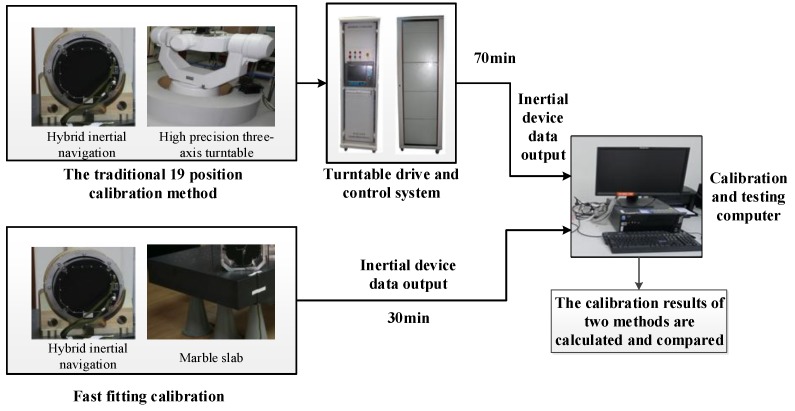
Experiment system composition.

First, the hybrid inertial navigation system is installed on a high-precision turntable (horizontal- and vertical-positioning accuracy of 2 arcsec. Then, the experiment is performed according to the positioning and calibration procedures of the 19-position calibration method. The hybrid inertial navigation system is in the turntable conditions after being heated. Second, we calibrate the hybrid inertial navigation system with the proposed method in this paper. We conduct a continuous dynamic self-calibration test according to the given experiment procedure. The hybrid inertial navigation system is placed on a plate on a stationary base and connected to an inertial navigation test equipment. We begin the calibration process according to the scheduled position and relying on the rotating frame structure of the hybrid inertial navigation system. The calibration parameters are calculated according to the output data. The experiment is continuously performed 3 times using the proposed method in this paper. The experiment system composition is shown in Figure 6. The results of the fast self-calibration of the three experiments and the calibration results of the 19-position method on a high-precision rotary turntable are compared to obtain a conclusion.

### 4.2. Experiment Data

The experiment data and calibration results are listed in Table 3.

### 4.3. Test Results Analysis

Under the condition that the hybrid three-axis rotating inertial navigation system is locked, the gyros and accelerometer parameters of the hybrid inertial navigation system are calibrated by 19-position calibration method based on the high-precision turntable. The calibration result is used as a standard value. Then, the continuous dynamic self-calibration of all parameters in the stationary base state is tested using the improved fast fitting calibration method in this paper and the calibration is performed three times. The results of the three groups are compared with the standard values and the maximum differences are calibrated as the accuracy to determine whether the precision requirements are met. The test results from the data are shown in Table 3: the gyro scale factor error is less than 1 ppm, the zero drift error does not exceed 0.00796°/h, the maximum gyroscopic installation error is less than 4.4″, the accelerometer scale factor error is less than 1 ppm, the accelerometer zero-bias error is less than 7.40 × 10^−6^ g and the accelerometer installation error does not exceed 3.2″. All parameters satisfy the requirements of calibration accuracy and the consistency of the calibration results of all calibration parameters is good. With the same precision, the calibration can be finished within 30 min using the fast fitting self-calibration method, whereas the 19-position calibration method with a high-precision rotary turntable requires almost 70 min. Thus, the proposed method in this paper can greatly reduce the calibration time and the calibration process is simple and easy to operate. The calibration equipment and processes are simplified by the method, which helps to eliminate the dependence on the high-precision turntable and improve the convenience of use.

Compared with the traditional 19-position calibration method and existing multi-position calibration method [26], the integration of the proposed fast fitting self-calibration method of the hybrid inertial navigation system in this paper has the following advantages:

(1) The method does not rely on the turntable accuracy; the calibration process is simple and easy to operate. The traditional calibration method relies on the high-precision turntable, that is, the calibration accuracy is greatly affected by the turntable accuracy and the calibration process and operation are complex. The proposed calibration method can be used to calibrate the inertial navigation on a marble slab.
(2) All parameters can be calibrated. Many calibration methods are only efficient for specific parameters but cannot achieve full parameter calibration, whereas the proposed method can be completed for all 21 error calibration parameters.
(3) The calibration time is short. With the precision of the traditional 19-position calibration method, the proposed self-calibration method reduces the calibration time from 70 min to 30 min and the calibration speed is fast.

## 5. Conclusions and Outlook

In this paper, to solve the calibration problem of hybrid inertial navigation systems (Inertial Navigation System), a fast dynamic self-calibration method for continuous dynamic under stationary conditions is proposed to satisfy engineering demands. Through a reasonable position arrangement, the error excitation is achieved during the position change and continuous overturn. Using the navigation speed and attitude error as navigation error observables, 21 error parameters are fully identified through the intermediate parameters. The simulation experiments and physical comparison tests show that the calibration accuracy of the calibration method does not depend on the precision of the turntable and installation accuracy and can reduce the calibration cost. The calibration sequence time is short and the identification parameter has high accuracy. The proposed method in this paper has high engineering application value.

## Figures and Tables

**Table 1:** Position arrangement.

Position	Pre-Rotation Attitude			Rotation Angle Rotation Axis	Post-Rotation Attitude		
	X	Y	Z		X	Y	Z
1	east	up	south	+90° Z	up	west	south
2	up	west	south	+90° Y	north	west	up
3	north	west	up	−90° X	north	down	west
4	north	down	west	−90° Y	west	down	south
5	west	down	south	−90° Y	south	down	east
6	south	down	east	−90° X	south	west	down
7	south	west	down	−90° Y	down	west	north
8	down	west	north	+90° Z	west	up	north
9	west	up	north				

**Table 2:** Simulation experimental data.

Parameter			Unit	Standard Values	Simulation Results	Precision
Gyros parameter repeatability	Scale factor	*W_gxx_*	ppm	50	50.67212	0.67212
		*W_gyy_*	ppm	50	50.93523	0.93523
		*W_gzz_*	ppm	50	49.43174	−0.56826
	Zero-drift	*D*_0*x*_	deg/h	3	3.00339	0.00339
		*D*_0*y*_	deg/h	3	2.99594	−0.00406
		*D*_0*z*_	deg/h	3	3.00177	0.00177
	Installation error	*W_gyx_*	arcsec	100	107.3	7.3
		*W_gzx_*	arcsec	100	102.7	2.7
		*W_gxy_*	arcsec	100	98.8	−1.2
		*W_gzy_*	arcsec	100	103.4	3.4
		*W_gxz_*	arcsec	100	106.6	6.6
		*W_gyz_*	arcsec	100	95.3	−4.7
Accelerometer parameter repeatability	Scale factor	*K_axx_*	ppm	30	30.75625	0.75625
		*K_ayy_*	ppm	30	29.53734	−0.46266
		*K_azz_*	ppm	30	30.12328	0.12328
	Zero bias	*E*_0*x*_	mg	3	3.00000614	6.14 × 10^−6^
		*E*_0*y*_	mg	3	2.99999663	−3.37 × 10^−6^
		*E*_0*z*_	mg	3	3.00000703	7.03 × 10^−6^
	Installation error	*K_azy_*	arcsec	50	53.6	3.6
		*K_axz_*	arcsec	50	55.2	5.2
		*K_ayz_*	arcsec	50	48.9	1.1

**Table 3:** Experiment data.

Parameters			Unit	Group One	Group Two	Group Three	19-Position Calibration Method	Precision (Maximum Error)
Gyros parameter repeatability	Scale factor	*W_gxx_*	ppm	50.60408	50.76921	50.25512	50.14445	0.62476
		*W_gyy_*	ppm	50.52607	50.42275	50.37525	50.38612	0.13995
		*W_gzz_*	ppm	50.65360	50.19866	50.26931	50.75971	0.56105
	Zero-drift	*D*_0*x*_	deg/h	−0.02387	−0.02651	−0.02492	−0.02777	0.0039
		*D_0y_*	deg/h	0.02557	0.01898	0.02422	0.02694	−0.00796
		*D*_0*z*_	deg/h	−0.18155	−0.17613	−0.17447	−0.18129	0.00682
	Installation error	*W_gyx_*	arcsec	401.3	396.3	397.6	398.7	2.6
		*W_gzx_*	arcsec	−112.3	−113.8	−115.1	−112.0	−3.1
		*W_gxy_*	arcsec	−80.6	−78.8	−78.3	−80.4	2.1
		*W_gzy_*	arcsec	−85.5	−89.7	−90.0	−85.7	−4.3
		*W_gxz_*	arcsec	431.0	427.5	427.0	431.4	−4.4
		*W_gyz_*	arcsec	−299.8	−297.1	−296.7	−300.6	3.9
Accelerometer parameter repeatability	Scale factor	*K_axx_*	ppm	30.14844	30.74637	30.39429	30.31055	0.43582
		*K_ayy_*	ppm	30.50457	30.47523	30.68148	30.52441	0.15707
		*K_azz_*	ppm	30.65408	30.30229	30.42456	30.61169	0.3094
	Zero bias	*E*_0*x*_	g	−0.0006743	−0.0006744	−0.0006698	−0.0006772	7.40 × 10^−6^
		*E*_0*y*_	g	0.0005472	0.0005463	0.0005476	0.0005468	8.00 × 10^−^^7^
		*E*_0*z*_	g	0.0007217	0.0007246	0.0007217	0.0007239	−2.20 × 10^−6^
	Installation error	*K_azy_*	arcsec	−7.5	−9.6	−9.0	−7.1	2.5
		*K_axz_*	arcsec	98.4	97.2	97.3	98.4	−1.2
		*K_ayz_*	arcsec	35.0	39.9	38.5	36.7	3.2
